# Discovery of Genes Underlying Cognitive Resilience in Individuals Predisposed to Alzheimer's Disease Risk

**DOI:** 10.1002/alz.084683

**Published:** 2025-01-09

**Authors:** Wei Tsai, Caitlin E. McNiff, Joseph S. Reddy, Xue Wang, Zachary Quicksall, Kwangsik Nho, Amy R Dunn, Mariet Allen, Michael G. Heckman, Yingxue Ren, Na Zhao, Kejal Kantarci, Michelle M. Mielke, Ronald C. Petersen, Catherine C. Kaczorowski, Minerva M. Carrasquillo, Andrew J. Saykin, Nilüfer Ertekin‐Taner

**Affiliations:** ^1^ Mayo Clinic, Jacksonville, FL USA; ^2^ Center for Computational Biology and Bioinformatics, Indiana University School of Medicine, Indianapolis, IN USA; ^3^ Department of Radiology and Imaging Sciences, Indiana University School of Medicine, Indianapolis, IN USA; ^4^ The Jackson Laboratory, Bar Harbor, ME USA; ^5^ Department of Radiology, Mayo Clinic, Rochester, MN USA; ^6^ Wake Forest University School of Medicine, Winston‐Salem, NC USA; ^7^ Department of Neurology, Mayo Clinic, Rochester, MN USA; ^8^ University of Michigan, Ann Arbor, MI USA; ^9^ Indiana University School of Medicine, Indianapolis, IN USA

## Abstract

**Background:**

Two main risk factors of Alzheimer’s disease (AD) are aging and APOE‐ε4. However, some individuals remain cognitively normal despite having these risk factors. They are considered “cognitively resilient”. This study aimed to identify molecular factors that confer cognitive resilience in APOE‐ε4 carriers ≥ 80 years of age and may serve as biomarkers.

**Method:**

We applied weighted gene co‐expression network analysis (WGCNA) to generate consensus co‐expression networks from blood of participants in two antemortem cohorts, the Mayo Clinic Study of Aging (MCSA, n=105), and the Alzheimer’s Disease Neuroimaging Initiative (ADNI, n=91), using RNA‐sequencing and microarray data, respectively. We associated these networks with resilience (resilient vs non‐resilient), cognitive endophenotypes and hippocampal volume. Preservation between consensus networks from blood and those derived from postmortem brain tissues of AD and control donors from AMP‐AD (n=1174) was evaluated. We validated the human findings in four AD mouse models. Finally, machine learning models were utilized to discriminate cases (AD+mild cognitive impairment (MCI)) from controls in MCSA, ADNI and ANMerge antemortem cohorts.

**Result:**

Four consensus networks were significantly correlated with a memory phenotype (logical memory delayed recall=LMDR) and hippocampal volume in both MCSA and ADNI. Among these, blood expression module M3 was most preserved with the brain transcriptome. M3 was enriched with NDUF hub genes that are involved in the mitochondrial respiratory chain. Expression levels of M3 and many blood NDUFs had significant associations with better LMDR and hippocampal volume. In brain, NDUFs were upregulated in controls compared to AD, and their expression levels were associated with better global cognition and decreased AD neuropathology. Many NDUFs were significantly downregulated in the hippocampus or cortex of AD mice compared to wild‐types. Lastly, models that included blood NDUFs improved diagnostic accuracy of AD+MCI compared to models that only included demographic and risk variables (age, sex, APOE‐ε4) in MCSA, ADNI and ANMerge. In MCSA and ADNI, adding NDUFs’ expression to models that included established blood biomarkers (Aβ42/40, ptau181, NFL) further improved diagnostic accuracy.

**Conclusion:**

Our results suggest that mitochondrial NDUFs are centrally‐linked peripheral molecular signatures that may be resilience factors against AD and serve as both therapeutic targets and novel diagnostic biomarkers.

